# Acceptance of clinical decision support system to prevent venous thromboembolism among nurses: an extension of the UTAUT model

**DOI:** 10.1186/s12911-022-01958-8

**Published:** 2022-08-19

**Authors:** Huixian Zha, Kouying Liu, Ting Tang, Yue-Heng Yin, Bei Dou, Ling Jiang, Hongyun Yan, Xingyue Tian, Rong Wang, Weiping Xie

**Affiliations:** 1 School of Nursing, Nanjing Medical University, Nanjing, 210000 People’s Republic of China; 2 Department of Nursing, Suzhou Municipal Hospital, Suzhou, Jiangsu 215008 People’s Republic of China; 3 The First Affiliated Hospital of Nanjing Medical University, Nanjing, 210029 People’s Republic of China; 4 Department of Nursing, The Second Affiliated Hospital of Suzhou University, Suzhou, Jiangsu 215000 People’s Republic of China

**Keywords:** Venous thromboembolism, Clinical decision support system, Nurses, Acceptance

## Abstract

**Background:**

Venous thromboembolism has been a major public health problem and caused a heavy disease burden. Venous thromboembolism clinical decision support system was proved to have a positive influence on the prevention and management of venous thromboembolism. As the direct users, nurses' acceptance of this system is of great importance to support the successful implementation of it. However, there are few relevant studies to investigate nurses' acceptance and the associated factors are still unclear.

**Objective:**

To investigate the determinant factors of nurses' acceptance of venous thromboembolism clinical decision support system with the modified Unified Theory of Acceptance and Use of Technology.

**Methods:**

We designed a questionnaire based on the modified Unified Theory of Acceptance and Use of Technology and then a cross-sectional survey was conducted among nurses in a tertiary hospital in Nanjing, China. Statistically, a Structural Equation Modeling -Partial Least Squares path modeling approach was applied to examine the research model.

**Results:**

A total of 1100 valid questionnaires were recycled. The modified model explained 74.7%, 83.0% and 86% of the variance in user satisfaction, behavioral intention and user behavior, respectively. The results showed that performance expectancy (β = 0.254, *p* = 0.000), social influence (β = 0.136, *p* = 0.047), facilitating conditions (β = 0.245, *p* = 0.000), self-efficacy (β = 0.121, *p* = 0.048) and user satisfaction (β = 0.193, *p* = 0.001) all had significant effects on nurses' intention. Although effort expectancy (β = 0.010, *p* = 0.785) did not have a direct effect on nurses' intention, it could indirectly influence nurses' intention with user satisfaction as the mediator (β = 0.296, *p* = 0.000). User behavior was significantly predicted by facilitating conditions (β = 0.298, *p* = 0.000) and user intention (β = 0.654, *p* = 0.001).

**Conclusion:**

The research enhances our understanding of the determinants of nurses' acceptance of venous thromboembolism clinical decision support system. Among these factors, performance expectancy was considered as the top priority. It highlights the importance of optimizing system performance to fit the users' needs. Generally, the findings in our research provide clinical technology designers and administrators with valuable information to better meet users' requirements and promote the implementation of venous thromboembolism clinical decision support system.

## Introduction

Venous thromboembolism (VTE), including pulmonary embolism (PE) and deep venous thrombosis (DVT), is estimated to be the third fatal cardiovascular event [1, 2]. It is a common complication among inpatients, which causes perioperative mortality and unexpected death [3]. During the past decade, the hospitalization rate of VTE has increased steadily from 3.2 to 17.5 per 100,000 population in China [4]. Moreover, the complications of VTE, like post-thrombotic syndrome, chronic thromboembolic pulmonary hypertension and hemorrhage seriously affect patients' quality of life and cause a heavy disease burden [5, 6].

VTE is considered as a preventable event [7, 8]. It is estimated that appropriate prophylaxis can reduce the relative risk of DVT and PTE by 50% and 66.7%, respectively [9, 10]. However, the current prophylaxis rate is poorly low [11]. A national, multi-center study [12] revealed that only 14.3% of inpatients at risk of VTE received some form of thromboprophylaxis, among which just 10.3% received appropriate prophylaxis recommended by guidelines. The data emphasized the insufficient management of VTE and showed the necessity to improve the clinical practice of medical staff.

Nurses as the largest cluster of medical staff, play a critical role in identifying inpatients at risk of VTE, implementing prophylaxis measures, and making clinical decisions [13–15]. Many hospitals have adopted VTE clinical decision support system (CDSS) to assist nurses to assess inpatients' individual risk and overcome the barriers in offering prophylaxis [16, 17]. The VTE CDSS is a computerized application system based on artificial intelligence and clinical information storage technology, which aims to realize the functions of risk stratification with the embedded risk assessment models (e.g. Paduwa, Caprini, Geneva), electronic alert reminder, priority preventive measures recommended, and the record of prevention process [17, 18]. Previous studies [17, 19] have shown the introduction of VTE CDSS can significantly increase the rate of adequate prophylaxis and then decrease the incidence of VTE.

However, it was shown that there existed an apparent mismatch between the benefits and adoption of CDSS among nurses [20, 21]. Nurses might become less likely to use the CDSS as they thought it brought them workload, work complexity and perceived threat to professional autonomy [20, 22]. Several studies explored the factors related to nursing staff's use of clinical technology concluded that the value of technology was determined by the appraisal of users [20, 23]. What's more, it has been reported that over 40% of information technology was failed or abandoned for the poor adoption of users [24, 25]. Hence, it is important to understand nurses' attitude and use intention toward the VTE CDSS and seek the influential factors to help engineers improve the design of the system and then extend the implementation and utilization of the system [26, 27]. Nowadays, in the field of technological nursing health care, little attention has been paid to the CDSS used by the nursing staff [25]. The factors that influence the nurses' acceptance of VTE CDSS are still unknown. The purpose of this research is to explore the acceptance of VTE CDSS among nurses and investigate the associated factors. This study was the first step in understanding the acceptance of nurses toward VTE CDSS. It is a relatively unresearched area, and thus the research we conduct is original and worthwhile.

## Theoretical framework and research hypothesis

### The original model

The Unified Theory of Acceptance and Use of Technology (UTAUT) is a widely used model to assess users' acceptance which was proposed by Venkatesh [28], based on eight related psychological and social theories/models. It contains four key constructs, which are effort expectancy (EE), performance expectancy (PE), social influence (SI) and facilitating conditions (FC) [28, 29]. According to the UTAUT, the first three constructs are the core determinants of the users' behavioral intention (BI) while the last construct directly influences the actual behavior use (UB) [28, 29].

UTAUT can explain 70% variance in technology use [30, 31]. Since introduction, it has been applied to explore the critical factors related to the prediction of users' intention and actual use of the technology in various health care settings, such as the health information system [32–34], mobile medical technology [35–37] and other clinical information systems [38, 39]. To our knowledge, UTAUT has not been applied to the field of VTE CDSS.

### The modified UTAUT and research hypothesis

Since UTAUT is a mature model, to provide a context-related understanding of technology acceptance, the theoretical model must be modified and tested for different technologies and different user groups under certain circumstances [40]. In antecedent researches, the UTAUT was used to be improved to contain key determinants to better investigate user' acceptance and usage of new systems [36, 41]. Therefore, our research improved UTAUT to include additional key determinants (user satisfaction and self-efficacy) based on antecedent researches and explore how these determinants affect the usage of the target system among nurses.

User satisfaction (US) is considered to be an important mediating variable influencing users' acceptance of the information technology [39, 42]. The Wixom and Todd (WT) model [43] combined US with technology acceptance, within the model, information satisfaction and system satisfaction represent a user's attitude toward the use intention of information technology. In addition, Abdrbo [44] believed that US can be measured with respect to the EE and PE constructs. Thus, in our research, we added the US variable and proposed the following hypotheses:

- H1: EE has a positive effect on nurses' US.
- H2: PE has a positive effect on nurses' US.
- H3: US has a positive effect on nurses' BI to use VTE CDSS.

Facilitating conditions (FC) measures whether there is an existence of the organizational and technical environment can help remove the barriers to implement CDSS [28, 29]. It was reported that when both EE and PE played a role, the effect of FC on the intention to use would not be significant and thus in the original model, the direct relationship between FC and BI was not included [28]. The review of 174 studies incorporating UTAUT [29] found that 48 of these original studies investigated the direct relationship between FC and BI, and 32 of those studies reported significant positive effects. It is worth noting that most of the included studies also found a significant effect of EE and PE [29, 45] which was contrary to Venkatesh's view [28]. So, it is necessary to re-examine the relationship between FC and BI, and then we proposed the following hypothesis:

- H4: FC has a positive effect on nurses' BI to use VTE CDSS.
  In 1977, Bandura [46] proposed the self-efficacy (SE) based on social cognitive theory. In the field of information technology, Davis [47] firstly discussed the influence of SE on students' intention to use a word processing software and found it was a vital determinant of the BI. Compeau [48] defined computer SE as an individual's judgment on their ability to use information technology, which means SE is an individual's level of confidence in using information technology to complete specific tasks. Several studies [49–51] demonstrated that computer SE played an essential role in predicting users' BI. Hence, we added the SE as a variable and proposed the following hypothesis:
- H5: SE has a positive effect on nurses' BI to use VTE CDSS.
  Additionally, we set up 5 hypotheses based on the original model:
- H6: EE has a positive effect on nurses' BI to use VTE CDSS.
- H7: PE has a positive effect on nurses' BI to use VTE CDSS.
- H8: SI has a positive effect on nurses' BI to use VTE CDSS.
- H9: FC has a positive effect on nurses' UB.
- H10: BI has a positive direct effect on nurses' UB.

**Fig. 1:**
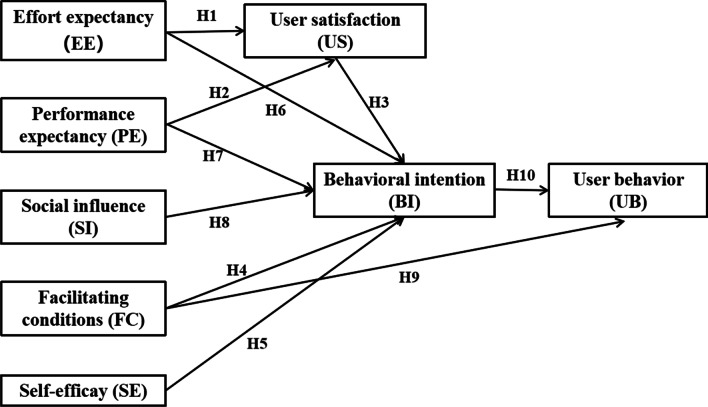
Research model

The modified UTAUT is presented in Fig. 1.

## Methods

All methods were carried out in accordance with relevant guidelines and regulations [8, 31–39].

### Research settings and participants

The target participants were recruited from the First Affiliated Hospital of Nanjing Medical University where the VTE CDSS has been implemented since 1 year ago. Registered nurses who took care of patients directly were qualified to participate in this research. Nurses in the internship and advanced training period were excluded.

### Research instrument

A questionnaire designed based on the previous research [32–39] was utilized to investigate the determinant factors of nurses' acceptance of VTE CCDSS. We did a brief introduction of the research and obtained informed consent from participants at the beginning. The questionnaire consisted of two parts. The first part was to collect basic information of participants, including gender, age, education, work ward, organizational position, length of the use, and training experience. No personal identification information of participants was involved. The second part included 29 questions covered 7 constructs based on the modified UTAUT model. Each item was evaluated through a 5-point Likert scale ranging from 1 (representing strongly disagree) to 5 (representing strongly agree).

The structured questionnaire was pilot tested for the expression and comprehension of each item. We recruited 10 nurses to conduct a preliminary investigation. Then the questionnaire was adapted to their feedback. The final questionnaire is presented in Table 1.

**Table 1:** Construct with its measurement items

Constructs	Corresponding Items
Effort expectancy (EE)	EE.1 The operating interface of VTE CDSS is clear
	EE.2 The contents of VTE CDSS are easy to understand and easy to use
	EE.3 Learning to use VTE CDSS is ease for me
	EE.4 It is easy and convenient for me to use VTE CDSS
	EE.5 It is easy for me to become skillful at using CDSS
Performance expectancy (PE)	PE.1 Using CDSS helps me dynamically assess and monitor the risk of VTE in patients
	PE.2 Using CDSS helps me make clinical decisions on VTE prevention (different measures according to the risk stratification)
	PE.3 Using CDSS helps me promote my work efficiency
	PE.4 Using CDSS helps me improve the quality of my work
Social influence (SI)	SI.1Hospital administrator (eg,. nursing department, special nursing unit, head nurse) think that I should use CDSS
	SI.2 Colleagues around me (including doctors) think that I should use CDSS
	SI.3 The surrounding leader or colleague who is a member of the hospital VTE group, think that i should use CDSS
	SI.4 The surrounding leader or colleague who participated in the design of the CDSS, think that i should use CDSS
Facilitating conditions (FC)	FC.1 I can get help from others when i have trouble in using the CDSS
	FC.2 During my work, CDSS works steadily
	FC.3 Hospital provides adequate training on the use of CDSS
	FC.4 I have the resources necessary to use CDSS
	FC.5 Hospital’s quality control results for CDSS promoted my use of the system
Self-efficacy (SE)	SE.1 I have a comprehensive knowledge of VTE prevention involved in CDSS
	SE.2 I am confident that I can use the system correctly
	SE.3I can skillfully use every medical information system in hospital
User satisfaction (US)	US.1 Information satisfaction: I am satisfied with the information provided by the modules of CDSS
	US.2 System satisfaction: I am satisfied with the overall operating process of CDSS
Behavior intention (BI)	BI.1 I intend to use CDSS in the future
	BI.2 I would like to continue to learn more about CDSS
	BI.3 I would like to recommend CDSS to others
User behavior (UB)	UB.1 I'm used to using CDSS
	UB.2 I will continue to use CDSS
	UB.3 I have recommended CDSS to others

### Data collection

The ethical approval of this study was obtained through the ethics committee of the First Affiliated Hospital of Nanjing Medical University (2020-SR-373). A cross-sectional survey was conducted from 25th September to 15th October 2021. The questionnaires were distributed by the medical and nursing departments through the Questionnaire Star Platform online.

We did a brief introduction of the research and obtained informed consent from participants at the beginning. The researchers predetermined that only participants completed all options in the questionnaires would they be able to submit it. In addition, participants could withdraw from the research at any time during the filling out process not until they clicked the submitted button, and these uncompleted questionnaires would be dismissed by the system. The completed questionnaires would be automatically sent back to our website (https://www.wjx.cn/weixinlogin.aspx). Any information participants provided were treated confidentially.

### Statistical analysis

We employed a Structural Equation Modeling (SEM)-Partial Least Squares (PLS) path modeling approach for data analysis [52]. SEM is a widely accepted paradigm to gauge the validity of theories with empirical data. SmartPLS software version3.0, one of the widely used software applications for PLS-SEM, was used to test and validate the proposed model [53].

The first part of the statistical analysis was to test the reliability and validity of the measurement model. The second part was to validate the associations between the hypothesized constructs and assess the fit indices of the model. In the first part, we assessed the outer loadings, cross-loadings and item-loadings of each item [54]. Then we tested the reliability of the measurement model with composite reliability (CR), the Average Variance Extracted (AVE), and Cronbach’s alpha [53, 55]. Thirdly, we checked the Variance Inflation Factor (VIF) for multicollinearity and determined whether the contribution of each individual indicator towards its construct was greater than 0 via outer weights. The last step in the first part was to measure whether the model fit was considered acceptable. The standardized root mean square residual (SRMR), squared Euclidean distance (d_ULS), geodesic distance (d_G), Normed Fit Index (NFI) and Chi^2^ were used to evaluate the fit of the model [56–58]. In the second stage, we tested the causal model and determined the effect sizes of the significant causal relations by f^2^. Survey data were exported from the website online database in the excel form and then imported into SmartPLS software for the analysis. *P* value ≤ 0.05 (two-tailed) was considered to be statistically significant.

## Results

### Descriptive statistical analysis

A total of 1100 questionnaires were recycled. The sample comprised 1081 women (98.27%) and 19 men (1.73%). The average age of nurses was 32.70 ± 6.978 and the length of working experience was 8.95 ± 7.608 year on average. The respondents worked in different departments in the hospital, for instance, respiratory medicine department, orthopedics department and intensive care unit. With respect to the education level of participants, 937 nurses (85.18%) had a bachelor’s degree, 11 nurses had a master or a doctor’s degree (1%) and only 152 nurses had a Junior College’s degree or below (13.82%). The descriptive statistics result is shown in Table 2. The mean scores of the constructs are shown in Table 3.

**Table 2:** Demographic characteristics of participants

Variable	Description	Frequency(n%)
Gender	man	19 (1.73%)
	woman	1081 (98.27%)
Age	32.70 ± 6.978	
Education	Junior College and below	152 (13.82%)
	Bachelor	937 (85.18%)
	Master or above	11 (1%)
Department	Surgical Department	342 (31.09%)
	Medical Department	306 (27.82%)
	Geriatric Department	149 (13.55%)
	Emergency Department	8 (0.73%)
	Maternal and Child Department	84 (7.64%)
	Intensive Care Unit	129 (11.73%)
	operating theatre	26 (2.36%)
	Medical technologic Department	43 (3.91%)
	Infectious Disease Department	13 (1.18%)
Length of working (year)	8.95 ± 7.608	
Professional title	Nurse	262 (23.82%)
	Nurse practitioner	502 (45.64%)
	Nurse-in-charge	263 (23.91%)
	Associate senior nurse	61(5.55%)
	Full senior nurse	12(1.09%)
Position	department head nurse	0
	Head nurse	69 (6.27%)
	Member of VTE special group	14 (1.27%)
	None	1017 (92.45%)
Length of CDSS usage	< 6 month	191 (17.36%)
	6 to 12 month	308 (28.00%)
	12 to 18 month	298 (27.09%)
	> 18 month	303 (27.55%)
Training frequency	Never	248 (22.55%)
	1 to 2 per year	703(63.91%)
	3 to 5 per year	104 (9.45%)
	> 5 per year	45 (4.09%)

**Table 3:** Mean scores of the constructs

Constructs	Standard deviations	Mean scores
EE	4.39 ± 0.672	4.39
PE	4.38 ± 0.669	4.38
SI	4.38 ± 0.690	4.34
FC	4.37 ± 0.660	4.37
SE	4.34 ± 0.672	4.34
US	4.36 ± 0.657	4.36
BI	4.43 ± 0.643	4.43
UB	4.37 ± 0.705	4.37

### The measurement model

In terms of the measurement model, we firstly assessed the outer loadings and cross-loadings of each item in 8 constructs. The results were depicted in Table 4, we found that the outer loadings were all over 0.7 (from 0.848 to 0.979), greater than the acceptable levels, so there was no need to remove any item. In addition, we assessed the cross-loadings through Table 4, all items load higher on the scale they were supposed to measure than on any other scale which ensures the discriminant validity of the measurement model. Secondly, we mainly examined the reliability of the measurement model by assessing composite reliability (CR), the Average Variance Extracted (AVE), and Cronbach’s alpha. As shown in Table 5, the result demonstrated that all constructs' Cronbach's alpha and CR are above 0.7, AVE values ranged from 0.847 to 0.928, greater than the threshold value of 0.50 [53]. Thirdly, we tested the variance inflation factor (VIF) values to determine if there was multicollinearity. The results were shown in Table 6, all VIF values were less than 10 (from 2.034 to 9.070), indicating that there was no multicollinearity problem between independent variables. The contribution of each item is greater than 0 via outer weights in Table 6, therefore all items were retained in then model. Lastly, the fit indexes of the research model was shown in Table 7, which indicate that the data collected fit well with the research model.

**Table 4:** Results of outer loadings and cross-loadings

Constructs	Item	EE	PE	SI	FC	SE	US	BI	UB
BI	BI.1	0.784	0.844	0.832	0.858	0.842	0.838	**0.964**	0.877
	BI.2	0.753	0.825	0.818	0.847	0.815	0.822	**0.966**	0.880
	BI.1	0.728	0.829	0.822	0.844	0.810	0.830	**0.960**	0.892
UB	BU.1	0.756	0.789	0.791	0.848	0.826	0.804	0.881	**0.952**
	BU.2	0.770	0.827	0.819	0.855	0.831	0.823	0.917	**0.956**
	BU.3	0.576	0.656	0.691	0.699	0.695	0.700	0.716	**0.848**
EE	EE.1	**0.938**	0.748	0.722	0.755	0.757	0.723	0.724	0.703
	EE.2	**0.948**	0.777	0.744	0.777	0.751	0.755	0.731	0.717
	EE.3	**0.955**	0.796	0.762	0.787	0.763	0.767	0.746	0.721
	EE.4	**0.961**	0.804	0.765	0.808	0.788	0.779	0.765	0.743
	EE.5	**0.917**	0.801	0.740	0.775	0.770	0.736	0.732	0.742
FC	FC.1	0.758	0.834	0.862	**0.908**	0.800	0.826	0.799	0.783
	FC.2	0.797	0.835	0.836	**0.924**	0.836	0.826	0.840	0.824
	FC.3	0.767	0.804	0.815	**0.929**	0.850	0.826	0.809	0.810
	FC.4	0.765	0.806	0.822	**0.944**	0.861	0.845	0.816	0.809
	FC.5	0.749	0.809	0.826	**0.934**	0.869	0.844	0.829	0.831
PE	PE.1	0.810	**0.933**	0.811	0.813	0.783	0.792	0.798	0.779
	PE.2	0.787	**0.944**	0.830	0.824	0.802	0.791	0.806	0.781
	PE.3	0.769	**0.936**	0.829	0.815	0.784	0.797	0.803	0.779
	PE.4	0.774	**0.936**	0.841	0.838	0.775	0.794	0.834	0.772
	PE.5	0.757	**0.933**	0.853	0.835	0.783	0.798	0.806	0.765
US	SA.1	0.770	0.826	0.830	0.877	0.884	**0.978**	0.836	0.819
	SA.2	0.789	0.833	0.832	0.880	0.884	**0.979**	0.850	0.835
SE	SE.1	0.761	0.805	0.814	0.897	**0.929**	0.838	0.813	0.817
	SE.2	0.782	0.785	0.789	0.852	**0.953**	0.845	0.815	0.816
	SE.3	0.740	0.771	0.766	0.808	**0.934**	0.862	0.775	0.775
SI	SI.1	0.775	0.841	**0.916**	0.842	0.790	0.795	0.819	0.794
	SI.2	0.725	0.849	**0.935**	0.821	0.767	0.781	0.790	0.767
	SI.3	0.745	0.831	**0.949**	0.851	0.799	0.805	0.809	0.799
	SI.4	0.714	0.809	**0.943**	0.843	0.794	0.799	0.783	0.769

**Table 5:** Construct Reliability and Validity

Constructs	Cronbach’s Alpha	Composite Reliability	Average Variance Extracted (AVE)
BI	0.961	0.975	0.928
UB	0.908	0.943	0.847
EE	0.969	0.976	0.891
FC	0.960	0.969	0.861
PE	0.965	0.973	0.877
US	0.955	0.978	0.957
SE	0.932	0.957	0.881
SI	0.953	0.966	0.876

**Table 6:** Variance Inflation Factor (VIF values) and Outer weights

Constructs	VIF	Outer weights	Constructs	VIF	Outer weights
BI.1	6.376	0.344	FC.5	5.317	0.220
BI.2	6.819	0.347	PE.1	5.309	0.212
BI.3	5.849	0.344	PE.2	6.313	0.213
BU.1	6.059	0.380	PE.3	5.473	0.213
BU.2	6.217	0.392	PE.4	5.529	0.217
BU.3	2.034	0.311	PE.5	5.184	0.214
EE.1	5.523	0.206	SA.1	6.050	0.507
EE.2	6.953	0.211	SA.2	6.050	0.515
EE.3	8.943	0.215	SE.1	3.438	0.360
EE.4	9.070	0.219	SE.2	4.807	0.361
EE.5	4.391	0.208	SE.3	3.898	0.344
FC.1	4.002	0.209	SI.1	3.708	0.273
FC.2	4.617	0.220	SI.2	4.623	0.264
FC.3	5.173	0.214	SI.3	6.810	0.270
FC.4	6.530	0.215	SI.4	6.435	0.261

**Table 7:** Fit indices of the research model

Fit	SRMR	d_ULS	d_G	NFI	Chi^2^
Research model	0.041	0.769	0.849	0.904	5049.868
Recommend value	< 0.1	–	–	0.9–1	–

### Hypothesis testing

**Fig. 2:**
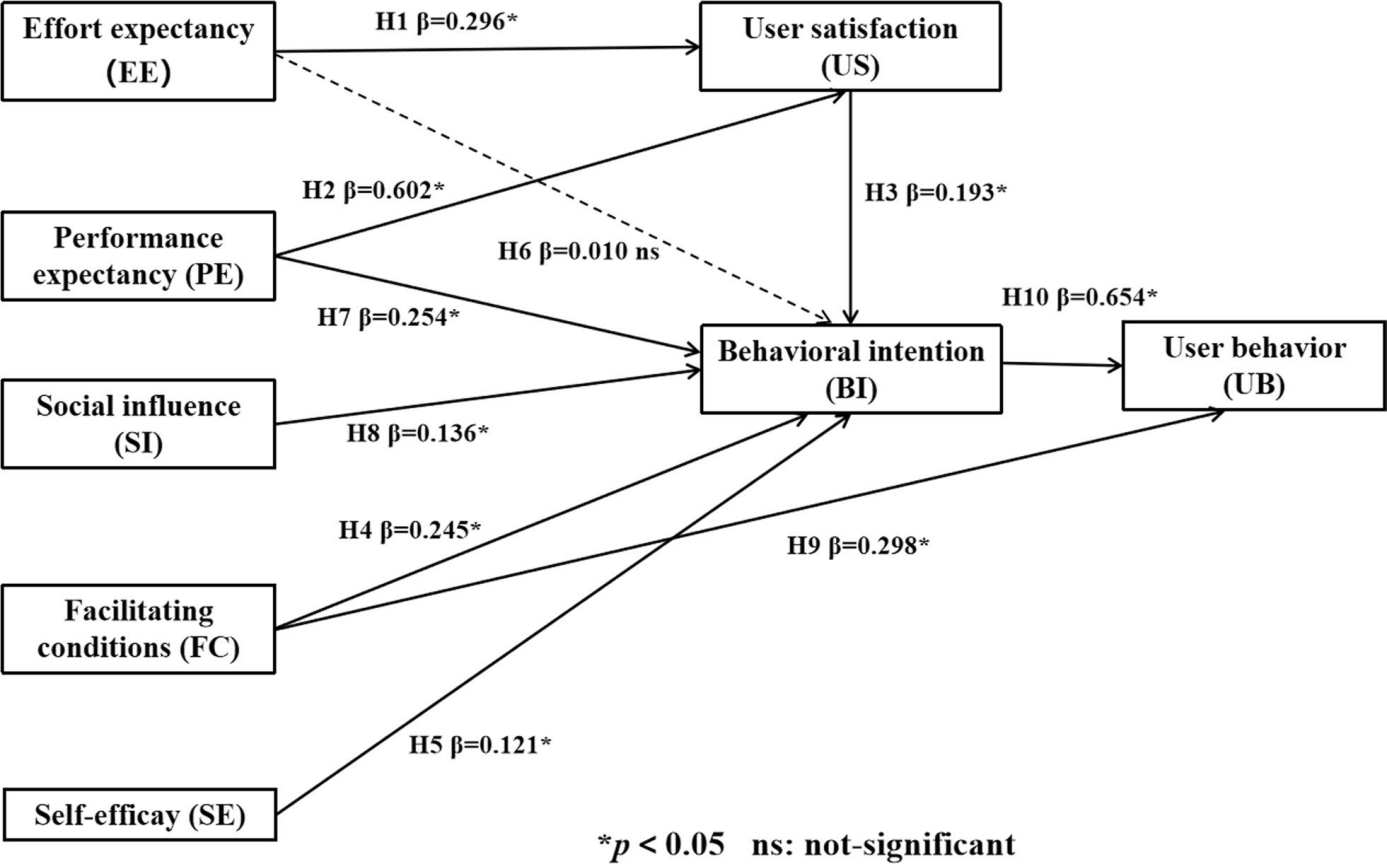
Causal model (results of hypothesis tests and path coefficient)

The structural model was then developed to investigate the relationships and the path coefficients between the constructs in the research model. We assessed the causal model via a bootstrapping procedure with 5000 bootstraps. The causal model is shown in Fig. 2.

The results showed that only one hypothesis, that EE has a positive effect on BI, was rejected (β = 0.010, *p* = 0.785). The relationship between PE and BI (β = 0.254, *p* = 0.000), SI and BI (β = 0.136, *p* = 0.047), FC and BI (β = 0.245, *p* = 0.000), SE and BI (β = 0.121, *p* = 0.048), US and BI (β = 0.193, *p* = 0.001) were all significant. Among these, PE is the strongest predictor of BI, with the path coefficients of 0.254. Although the relationship between EE and BI is unsupported, it was statistically shown that EE (β = 0.296, *p* = 0.000) indirectly influenced nurses' intention with the US as the mediator. And PE (β = 0.602, *p* = 0.000) played the same indirect role on BI as EE did. Additionally, UB was significantly predicted by FC (β = 0.298, p = 0.000) and BI (β = 0.654, p = 0.001). We determined the effect size (f^2^) of the significant relations in the model, these scores are as follows: H1: f^2^ = 0.106 (small effect size), H2: f^2^ = 0.440 (large effect size), H3: f^2^ = 0.031 (small effect size), H4: f^2^ = 0.035 (small effect size), H5: f^2^ = 0.011 (small effect size), H7: f^2^ = 0.057 (small effect size), H8: f^2^ = 0.016 (small effect size), H9: f^2^ = 0.140 (medium effect size), H10: f^2^ = 0.675 (large effect size).

Partial least squares path modeling revealed that the variance of US explained by the modified UTAUT model was 74.7%. The variance of BI explained by the modified UTAUT model was 83.0% which is higher than the original test result by the Venkatesh [28] (the variance of BI explained by the model was almost 70%). Additionally, FC and BI directly influenced the UB and all constructs in the model explained 86.0% variance of the UB.

## Discussion

This research utilized UTAUT as a theoretical basis and integrated related variables to investigate the influential factors associated with the acceptance of VTE CDSS among nurses. The aforementioned statistical results all showed the effectiveness of the modified UTAUT model in predicting nurses' acceptance of VTE CDSS. The findings of the research indicated that: 1. EE and PE with US serving as a mediator significantly affected nurses' BI to use VTE CDSS; 2. nurses' intention to use CDSS was associated with their perceived ease and usefulness of use, their significant others concerning about VTE CDSS, their perception about the organizational and technical environment, their confidence and satisfaction over the usage process; 3. lastly, FC and BI had a significant direct effect on nurses' actual use of VTE CDSS. As we know, this is the first study to investigate the nurses' acceptance of VTE CDSS.

PE was the strongest factor in predicting nurses' intention to use VTE CDSS. This result is consistent with the antecedent researches [36, 38]. CDSS can positively improve nurses' knowledge and enhance their awareness of VTE prevention as it provides instruction as soon as patients' information is recorded into the system [23, 25]. The embedded electronic alert can also remind nurses to reassess the VTE risk within the specified time or when the patients' condition changes [10, 17]. Porat [55] stated that medical staff could give patients more personalized education while using the CDSS. What's more, previous research [59–61] indicated that CDSS could help improve evidence-based clinical practice overall by improving the medical staff's performance and work efficiency. However, there still exist research suggesting that medical staff considered it as a threat to their clinical autonomy since they used to depend on their experience and preference to provide patient care [62, 63]. They also reported that it took them too much time to document in the CDSS and thus their attention to patients decreased [25, 59, 63]. During the literature review of VTE CDSS, we also noticed a phenomenon called alert fatigue [64]. This means users have a tendency to ignore or over-ride the triggered tool due to the frequent alerts [18, 64]. In general, most current studies showed that the advantages of the CDSS outweigh the disadvantages [18, 62–64]. Since all users tend to agree PE construct is of great importance in the nurses' intention to use and maintain use in the future [36, 54], the VTE CDSS still needs continuous improvement to remove the barriers of usage and increase its perceived usefulness.

However, our study did not find a significant relationship between EE and BI which may be apparently surprising, since EE is a core construct of UTAUT and is considered as a determinant of BI based on many antecedent empirical tests [28–30], especially in the elderly group [54]. Several studies also found a similar result [37, 46, 47], and one possible explanation for this is that our VTE CDSS is easy to use and thus the effect of EE is not salient [47]. Another explanation is our participants were relatively young (with the mean age of 30) and well educated (85.18% with a bachelor's degree), so using an information system may not be a complicated job. Nonetheless, these statistical results do not mean that the EE construct does not help explain the adoption of VTE CDSS. EE has an indirect effect on BI, and this effect is mediated by users' satisfaction. Both PE and EE correlate with users' satisfaction (US), and then the US directly influences BI. Ray [65] have ever emphasized the importance of user-centered design CDSS, which means users could easily learn without training and use it correctly. To promote users' satisfaction, the system must be user friendly and useful with both satisfying information and system quality [62, 65]. With increased satisfaction, the users' intention will be enhanced [62, 63, 65].

SI had a significant relationship with BI. A survey conducted by Lu [32] found a prominent effect of SI on the adoption of hospital information system among nurses. What the two studies have in common is that both hospitals have encouraged and supported the nurses to adopt the systems [32]. It is assumed that medical staff refused to use the system for the lack of policy regulations by the hospital administrators [66]. Hence, the regulatory issues are vital to the nurses' adoption of the VTE CDSS [33, 66]. Additionally, the VTE CDSS generated documentation can be imported into the clinical note and the successor can view the previous worker's documentation, which is conducive to hand-over work and fully grasp the patient's condition [67]. Gradually, consistent consensus on the use of the system was achieved among nurses and they became more familiar with it.

The results established the link between FC with BI and UB. FC is considered as a highly rated requirement by all medical staff [32, 67]. These organizational and technical factors are significant for the nurses, for instance, whether the infrastructure is sufficient to support the use of CDSS (e.g. network with good signals, fully equipped computers) or whether the users receive adequate training [18, 66]. As we can see, among our participants, there still left 22.55% of participants reported that they had never received any training. What's more, among the training people, more than half stated that they only received 1 or 2 times training. Both hospital administrators and manufacturers should jointly provide nurses with adequate training and continuous assistance services to support the implementation of VTE CDSS [18, 32, 66].

The significant relationship between BI and SE highlighted the importance of SE. SE here refers to the computer self-efficacy which has been proved to play an essential role in predicting users' intention and behavior when using computer [33, 49]. It was found to be associated with the computer literacy and computer anxiety [48, 49]. People exhibited higher computer anxiety tend to show lower computer literacy, which means the two concepts play a counterproductive role [48–50]. It is believed that the use of CDSS is dependent on computer literacy and lack of technical proficiency can be hindering [18]. Computer anxiety is an unpleasant feeling which contains negative emotional states during the interaction with computer [48, 49]. Women exhibit lower computer literacy and higher computer anxiety than men, so women ultimately have lower computer SE [53]. In our survey, women occupied 98.27% of all participants, so we should pay more attention to such users' computer SE. Several studies indicated that the increased level of education would enhance the level of SE [67]. Providing adequate and related training courses on the new technology will help nurses decrease their anxiety and increase their confidence to use the system [67, 68]. For better acceptance of VTE CDSS, they should not only improve their knowledge of the system, but also increase their professional knowledge since many modules in VTE CDSS need their own expertise.

This study has the following implications for the future research: first, the context-related determinants (including EE, PE, SI, FC, SE and US) provided in our research should be considered to understand users behavior intention and actual behavior. Second, our research highlights the importance of performance expectancy, however, the “setting of electronic alerts” and the “threat to clinical autonomy” come after the usage of VTE CDSS are still unclear and should be further explored in the future research. In the other hand, this study also has implications for clinical practice. Under the trend of medial information technology development, more and more clinical information technology systems are being put into clinical use. The main challenges for technology are the users’ acceptance and the successful integration of systems with the entire clinical workspace. Thus, the healthcare organizations must pay attention to users’ needs and their acceptance of the new technology. The ideas and methods of our research can provide reference for healthcare organizations. In addition, our results can help developers of the VTE CDSS know what is considered to be important in using the system by nurses, and then they will be able to develop and improve the system which fits into the daily clinical practice of nurses perfectly.

There are some limitations of this study. First, our study is a cross-sectional survey, therefore we can only determine association but not establish a causal relationship between the variables and outcomes. Second, our study used a web-based survey, all constructs were measured as self-report measures. The participants completed the questionnaires with respect to their working experiences and perceptions. This may lead to common variance bias and the results must be interpreted with caution. Third, since the CDSS was already implemented one year ago in the hospital, we did not have access to individual usage. The data of usage was recorded in the hospital manage system in each department as a unit but not an individual basis, so user behavior was measured by means of the questionnaire, instead of by means of how the participants used the implemented CDSS. Fourth, nowadays in China, the VTE CDSS has not been widely used. Implementing a CDSS is a complex undertaking of successive stage and the length of use is inconsistent among various hospitals and departments, so this survey was carried out in a single site. We did not take the characteristic differences between different hospitals and different types of VTE CDSS into account. Hence, it is necessary to conduct further studies covering more hospitals in more regions in the future.

## Conclusion

Our study supports the use of the modified UTAUT to predict the factors associated with nurses' acceptance of VTE CDSS. This research makes significant contributions to the VTE CDSS implementation and thromboprophylaxis research. The findings indicated that performance expectancy is the most important determinant of nurses' intention to use CDSS. Users' needs and expectations need to be fully considered by the developers during the development and improvement of VTE CDSS. Besides, hospital administrators should pay more attention to users’ acceptance of the system, the ideas and methods of our research can provide reference for healthcare organizations. Nowadays, CDSS is in its initial stage in developing country. Future research can assess the users' acceptance of the system in a quantitative way and if the users' acceptance do not reach the predefined threshold, the system should be subjective to the adaptive redesign.

## Data Availability

All data generated or analysed during this study are included in this published article.

## Abbreviations

VTE: Venous thromboembolism
PE: Pulmonary embolism
DVT: Deep venous thrombosis
CDSS: Clinical decision support system
UTAUT: Unified Theory of Acceptance and Use of Technology
EE: Effort expectancy
PE: Performance expectancy
SI: Social influence
FC: Facilitating conditions
BI: Behavioral intention
UB: Behavior use
US: User satisfaction
SE: Self-efficacy
SEM: Structural Equation Modeling
PLS: Partial Least Squares
CR: Composite reliability
AVE: Average variance extracted
VIF: Variance inflation factor
